# Age-related changes in oscillatory power affect motor action

**DOI:** 10.1371/journal.pone.0187911

**Published:** 2017-11-27

**Authors:** Liqing Liu, Nils Rosjat, Svitlana Popovych, Bin A. Wang, Azamat Yeldesbay, Tibor I. Toth, Shivakumar Viswanathan, Christian B. Grefkes, Gereon R. Fink, Silvia Daun

**Affiliations:** 1 Heisenberg Research Group of Computational Neuroscience—Modeling Neural Network Function, Department of Animal Physiology, Institute of Zoology, University of Cologne, Cologne, Germany; 2 Cognitive Neuroscience, Institute of Neuroscience and Medicine (INM-3), Research Centre Jülich, Jülich, Germany; 3 Department of Neurology, University Hospital Cologne, Cologne, Germany; Universitair Medisch Centrum Groningen, NETHERLANDS

## Abstract

With increasing age cognitive performance slows down. This includes cognitive processes essential for motor performance. Additionally, performance of motor tasks becomes less accurate. The objective of the present study was to identify general neural correlates underlying age-related behavioral slowing and the reduction in motor task accuracy. To this end, we continuously recorded EEG activity from 18 younger and 24 older right-handed healthy participants while they were performing a simple finger tapping task. We analyzed the EEG records with respect to local changes in amplitude (power spectrum) as well as phase locking between the two age groups. We found differences between younger and older subjects in the amplitude of post-movement synchronization in the β band of the sensory-motor and medial prefrontal cortex (mPFC). This post-movement β amplitude was significantly reduced in older subjects. Moreover, it positively correlated with the accuracy with which subjects performed the motor task at the electrode FCz, which detects activity of the mPFC and the supplementary motor area. In contrast, we found no correlation between the accurate timing of local neural activity, i.e. phase locking in the δ-θ frequency band, with the reaction and movement time or the accuracy with which the motor task was performed. Our results show that only post-movement β amplitude and not δ-θ phase locking is involved in the control of movement accuracy. The decreased post-movement β amplitude in the mPFC of older subjects hints at an impaired deactivation of this area, which may affect the cognitive control of stimulus-induced motor tasks and thereby motor output.

## Introduction

When people age, they perform complex tasks more slowly, and often, less accurately than they once did [1]. This includes even simple motor tasks. For example, aging leads to an increased variability in movement execution, as well as to a progressive slow-down in response to external stimuli [2, 3]. Moreover, older adults exhibit a reduced accuracy in the performance of visually guided movements [4]. The variability of age-related movement and the slow-down in behavior have complex origins, being a combination of neural degeneration as well as degeneration of the muscles and effectors [5]. To better characterize the neural origins of age-related behavioral changes, we, in the present study, made use of electroencephalograms (EEG) to investigate whether age-related changes might affect some functions of the cortical motor system more strongly than others.

The cortical motor system is associated with a variety of characteristic neural oscillations over a broad range of frequencies [6]. In general, neural oscillations in the frequency domain can be described by their amplitude and phase. While the former represents the power of the oscillation, the latter represents the oscillation’s timing with regard to a reference point in time [7–9]. Since these two properties of neural oscillations–power and phase–have been associated with different aspects of motor function, we conjectured that they could help identify and characterize the complex motoric changes that occur during normal aging.

Phase locking of neural activity is a basic mechanism of neural synchronization, which is assumed to enable communication between spatially separated neural populations and to be a representation of coordinated information processing [9–11]. Both phase resetting or locking of ongoing activity and event-related activation of neural assemblies are involved in the generation of event-related potentials (ERPs) [10, 12, 13]. In a previous study, we identified a phase-related signature of movement. When younger healthy subjects performed a simple binary choice task that required movements of either the left or the right index finger, we found that oscillations in the δ-θ frequency band showed a characteristic phase locking at electrodes contralateral to the moving hand, irrespective of whether the movement was internally or externally triggered [9]. The neural generators of the lateralized phase locking in the δ-θ frequency band are motoric but not exactly identified (as reported in [9]). Nonetheless, it proved to become a convenient tool to assess motor timing.

**Fig 1:**
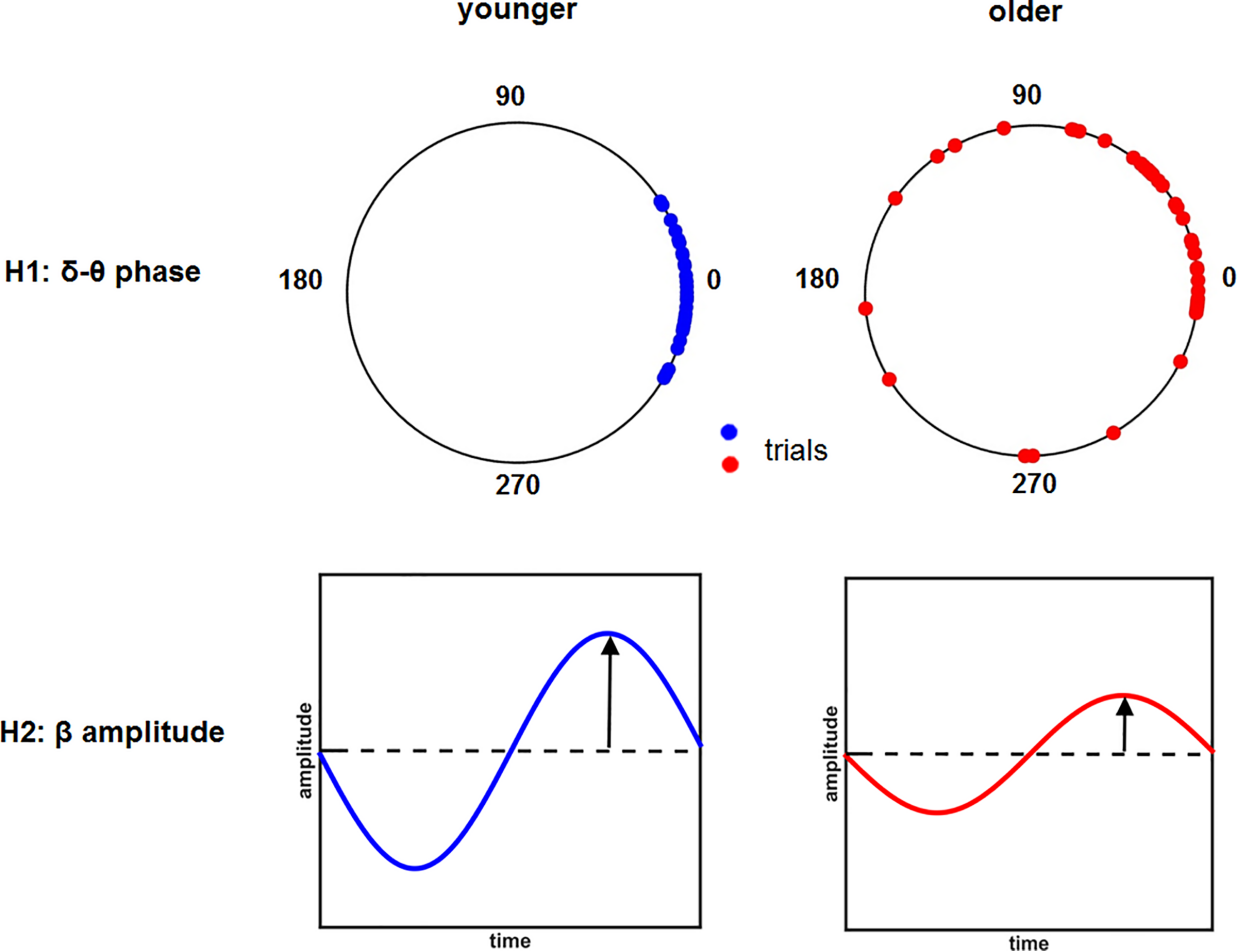
Illustration of our hypothesis that the older subjects may show a decreased δ-θ phase locking index (H1) or β amplitude (H2) since the variability of the motor performance increases with age. In the upper panel, the dots represent the phase distribution in the δ-θ frequency band across trials in both younger (YS, blue) and older (OS, red) participants. In the lower panels, amplitude changes in the β frequency band (ERD and ERS) are displayed as they may appear in younger (YS, blue) and older (OS, red) subjects.

Phase locking was quantified by using the phase locking index (PLI) (cf. Material and methods of this manuscript). PLI is large if oscillations are reset at nearly the same oscillatory phase across trials. Thus, a small PLI implies that the movement-related phase resetting does not consistently occur at the same phase across trials and therefore reflects a weak neural synchronization. Since movement-related PLI in the δ-θ frequency band is a measure of variance of the timing in the cortical areas, we hypothesized that if age-related movement variability were linked to variability of cortical oscillations in the motor areas, then PLI in the δ-θ frequency band, being large in younger individuals (cf. [9]), would show a relative decrease in older individuals (see Fig 1A).

On the other hand, the cortical motor system has long been associated with power changes, particularly in the α and β frequency bands [14]. Unlike phase changes, the ones in the power spectrum have been linked to the proportion of neurons that fire at the same time (i.e., in 'synchrony'). The more neurons fire concurrently, the greater the reduction of the power at low frequencies. Another possibility we considered here is that the age-related changes might result in insufficient cortical inhibition [15] or in loss of cortical motor neurons [16]. Based on this observation, we conjectured that the β amplitude would decrease in the older subjects (see Fig 1B).

Using the previously established experimental paradigm described in [9], we continuously recorded EEG activity from older healthy participants while they were performing the same simple, internally or externally driven motor task, in the same experimental conditions, as the younger ones. We then analyzed these data and compared the results to those previously obtained in younger healthy subjects.

Our results show that phase locking in the δ-θ frequency band is a general neural mechanism accompanying movement execution. However, it appears to be independent of the age of the subjects, hence it does not reflect age-related deficits in motor behavior. In contrast, post-movement ERS in the β band is diminished in older subjects and related to age-dependent deficits in motor accuracy, measured as the extent and frequency of errors made.

## Material and methods

### Participants

Thirty-one older (15 females; age range: 60–78 years) healthy individuals were recruited from the subject database of the Research Center Juelich. Data from 3 subjects had to be excluded due to poor performance or erroneous movements (e.g., multiple taps). Data from 4 more participants were excluded because of poor quality of the records (see EEG pre-processing section). Hence, data from 24 older subjects were used for further analysis and later compared with data from 18 younger healthy subjects (acquired in [9]). Experimental set-up and conditions were identical in both studies.

All participants gave written informed consent prior to their participation. They were all right-handed as defined by the Edinburgh Handedness Inventory [17]. All subjects had normal or corrected-to-normal vision and no medical history of neurological, psychiatric, or orthopedic diseases. The study was approved by the Ethics Committee of the Medical Faculty of the University of Cologne.

### Experimental task

The subjects performed a finger tapping task in three different conditions: self-initiated tapping, visually-cued tapping, and vision-only (i.e., no finger tapping). The latter condition served as control [9].

In the self-initiated tapping condition, subjects were asked to voluntarily press a button with the left or right index finger while adhering to the following constraints that were necessary for technical reasons (e.g., temporal separation of the EEG events; response bias): 1) The duration between two button presses should be 4 to 8 seconds. 2) The participants should roughly balance the number of presses performed by the left and right index fingers. 3) The subjects were instructed to avoid explicit counting when performing the task. In order to restrict eye movements, subjects were required to fixate on a white cross presented against a black background in the center of a computer monitor.

In the visually-cued tapping condition, red arrows directed to the left or to the right were presented with inter-stimulus intervals (ISI) of varying length in the center of the computer screen. The length of ISI was determined from the interval lengths between the motor actions observed in the self-initiated condition. The participants were instructed to respond with the respective index finger (left or right), i.e., to press a button with the left or right index finger, as soon as an arrow occurred.

In the vision-only condition, red arrows were presented on the screen in the same way as in the visually-cued tapping condition but participants were instructed only to look at the screen without performing the motor task (or an imaginary movement).

A complete experiment consisted of 4 runs (16 blocks in total) and lasted about 70 minutes (each run lasted approximately 17 minutes). In each block, all three conditions were presented, starting with the self-initiated tapping condition.

The order of occurrence of the visually-cued and the vision-only tapping condition was randomized (cf. [9]).

### Experimental procedure

The participants were seated comfortably in front of a computer monitor in a dimmed EEG-chamber with their head kept in a static position by means of a chin-rest. The distance of their eyes from the presentation screen was approximately 70 cm. The keyboard of which the keys had to be pressed was placed under the board of a table in front of the participants. It was hidden from direct viewing in order to prevent involuntary eye movements to the hands. EEG was recorded while the subjects were performing the tasks described above. In addition, accelerometers were firmly attached to the dorsal tip of both index fingers with an adhesive tape, in order to identify the onset of the movement. During the EEG recording, the participants were instructed to avoid eye blinks, swallowing, or movements other than tapping with their index fingers. During the whole experiment, a video camera was used to monitor that the participants followed the instructions and stayed awake. To avoid any misunderstanding as to the experimental condition to be performed, or to a forbidden pattern produced by the participants in the self-initiated tapping condition, the experimenter checked their performance from outside the EEG-chamber during the EEG recording using the software Brainvision recorder (Brainproduct, Munich, Germany) (cf. [9]).

### Data acquisition

In the experiments, continuous EEG (actiCAP, Brain Products GmbH, Munich, Germany) was recorded using 64 electrodes covering the whole scalp according to the international 10–20 system [18]. The reference electrode was placed at the left earlobe, since we expected this location not to be involved in visual processing, motor planning, or execution. Bilateral horizontal and left vertical electro-oculograms (EOG) were recorded by three electrodes (FT9, FT10, and TP10, according to the international 10–20 system). Two of them were placed on the outer canthi of the eyes and one was placed under the left eye. Electrode impedances were measured before and after the recording and kept ≤15 kΩ. The EEG records were digitized at a sampling rate of 2500 Hz and filtered with a 0.87 Hz high pass and 500 Hz low pass filter.

During recording, the acceleration sensors captured the finger movements in three dimensions. Movement kinematics were later used to identify the onset of the movements. The derivatives of the three components of the acceleration signal were computed, and then the (Euclidean) length of this vector was calculated at each time point. The time-series were then smoothed, rescaled, and a threshold was set to identify the earliest time point in a 125 ms window prior to each button press in which a monotonic increase in acceleration rate took place. This time point was defined as the movement onset. All responses in which the movement onset could not be detected unambiguously were excluded from further analysis. Based on these accelerometer data, we defined the movement time as the time that elapsed from movement onset to the button press in both the visually-cued and the self-initiated tapping condition.

An additional behavioral measure of task performance, the accuracy rate, was defined, in the visually-cued tapping condition, as the ratio of the number of correct trials to the total amount of trials. Wrong responses, i.e., responses where the buttons were pressed with the wrong index finger, or missed responses were defined as incorrect trials in the visually-cued tapping condition. In the self-initiated tapping condition, no equivalent behavioral measure could be defined, since it was difficult to determine what constituted an error in such an experimental condition. Also, only in the visually-cued condition, the reaction time was defined as the time that elapsed from the presentation of the stimulus to the button press.

### EEG data preprocessing

The EEG data were preprocessed in EEGLAB [19], which operates in the MATLAB (Mathworks, Natick, MA) environment. After down-sampling of the EEG records, high pass (0.5Hz) and low pass filtering (48 Hz) were, separately, performed on the continuous EEG data. Next, the EEG data were visually inspected for paroxysmal and muscular artifacts not related to eye-blinks. Then, the artifacts in the EEG records marked during visual inspection were excluded from further analysis. Responses in which the time interval between two button presses was shorter than 4 seconds in the self-initiated tapping condition and responses with a reaction time longer than 1 second in the visually-cued tapping condition were marked as invalid trials.

Next, the continuous data were divided into epochs, from -2500 ms to 1500 ms in the self-initiated tapping condition, and from -2000 ms to 1500 ms in the visually-cued tapping condition. For further details, cf. [9]. After the epoching procedure, invalid trials were discarded. This led to an exclusion of 12% of the data. Bad channels were detected automatically by EEGLAB based on channel statistics with a trim percentage of 5. Data from bad channels were removed and re-interposited by means of spline interpolation using data from the neighboring channels. Channel data with eye blinking and other artifacts different from neural activity were identified and removed using independent component analysis (ICA). Finally, we used the first 1000 ms of each epoch as baseline and performed a baseline correction for all epochs.

### Time-frequency decomposition and phase-locking analysis

The time-frequency and phase-locking analysis were performed on the signals of all electrodes using SPM 12 (http://www.fil.ion.ucl.ac.uk/spm/) in the MATLAB environment (The MathWorks Inc., Massachusetts, USA). As it is known, EEG has high time resolution (up to ms) and a relative low spatial resolution. In order to improve the spatial resolution and to eliminate the influence of the distortion of the reference electrode, the small Laplacian [20] was computed at each of the electrodes, except for the boundary ones, using SPM 12.

The artifact-free and epoched EEG data were transformed into the time-frequency domain and decomposed into amplitude and phase components using complex Morlet transformation with a Morlet wavelet factor of five. We analyzed the data in the frequency band from 1Hz to 48Hz with a resolution of 1Hz. Having computed the logarithm of the ratio of the amplitude to the baseline, we averaged the amplitude normalized thus for all trials in each condition and for every subject.

Using the phase information, we calculated the phase-locking index (PLI) [21, 22] for each subject. The PLI is defined as follows:

$$PLI\;\left(t,f\right)=\left|\frac{1}{N}\sum_{k=1}^{N}\text{exp}\left(i\varphi_{k}\left(t,f\right)\right)\right|$$

where N is the number of trials, and φ_k_ is the phase of trial k at time t and at a given frequency f; i is the imaginary unit: i^2^ = −1. PLI is a measure of similarity of the phases of a signal over many repetitions. It can range from 0 to 1, where 1 means identical phase of the signal across all trials and 0 indicates uniformly distributed random phases.

As we were interested in analyzing differences in both the PLI and the amplitude in the groups of younger and older participants, respectively, we restricted our further analysis to the EEG electrodes that exhibited significant phase locking compared to the baseline. For each of these electrodes we calculated and averaged the amplitude and PLI over frequencies in the different frequency bands, i.e., in the δ-θ (2-7Hz), α (8-12Hz), β (13-30Hz), and low γ (31-48Hz).

### Statistical analysis

For the statistical analysis of the behavioral data, a three-way (hands x conditions x groups) analysis of variance (ANOVA) as well as a two-sample t-test was used to detect differences between the two age groups in movement times in both the self-initiated and visually-cued tapping condition, as well as in reaction times in the visually-cued tapping condition. Differences between age groups in accuracy rates in the visually-cued tapping condition were tested with a two-sample t-test, since left and right index finger tappings were not separated.

Statistical maps showing the phase-locking index at all 61 electrodes in the self-initiated as well as in the visually-cued tapping condition were constructed according to the statistical procedure described in [9].

There are several methods that can be used for statistical analysis of PLI [23]. In our study, we used two sample t-test and ANOVA on epoched data. To test for significant differences in PLI and amplitude between the two age groups, we calculated the average PLI value over the δ-θ frequency band as well as the average amplitude value over the four frequency bands (δ-θ, α, β and γ) separately and performed paired t-tests pointwise on each of the average PLI and amplitude curves at every time point (from -1400 ms to 1400 ms) at each electrode of interest for both conditions, i.e., self-initiated and visually-cued tapping, and for both hands in the different frequency bands, at a significance level of 0.05. For multiple comparisons, FDR correction (for time points, electrodes, conditions, and hands) was applied.

To confirm whether the differences between the younger and older participants were statistically robust, we used a three-way (hands x group x electrodes) ANOVA on the averaged amplitude over the time period that shows difference between the two groups.

One-sample Kolmogorov-Smirnov (K-S) test was applied in order to check whether the data were normally distributed before applying the t-test.

### Correlation analysis

To test for correlations between behavioral performance and electrophysiological data, Pearson correlations were computed between the mean β amplitude and the reaction and movement time as well as between the mean β amplitude and the accuracy rate. These calculations were performed for both groups (younger and older participants). Results were corrected for multiple comparisons (FDR, number of electrodes).

## Results

### Behavioral results

We first tested whether there were significant differences in motor performance between older (OS) and younger subjects (YS) with respect to movement time (MT), reaction time (RT) and accuracy rate. The MT, RT and accuracy rate was normally distributed.

For the reaction time and movement time, a three-way (hands x conditions x groups) ANOVA was used. There was a significant main effect of the hands ((F (1, 120) = 7.94, p = 0.0054)) and groups ((F (1, 40) = 31.92, p < 0.0001)). No interaction effect was found.

**Fig 2:**
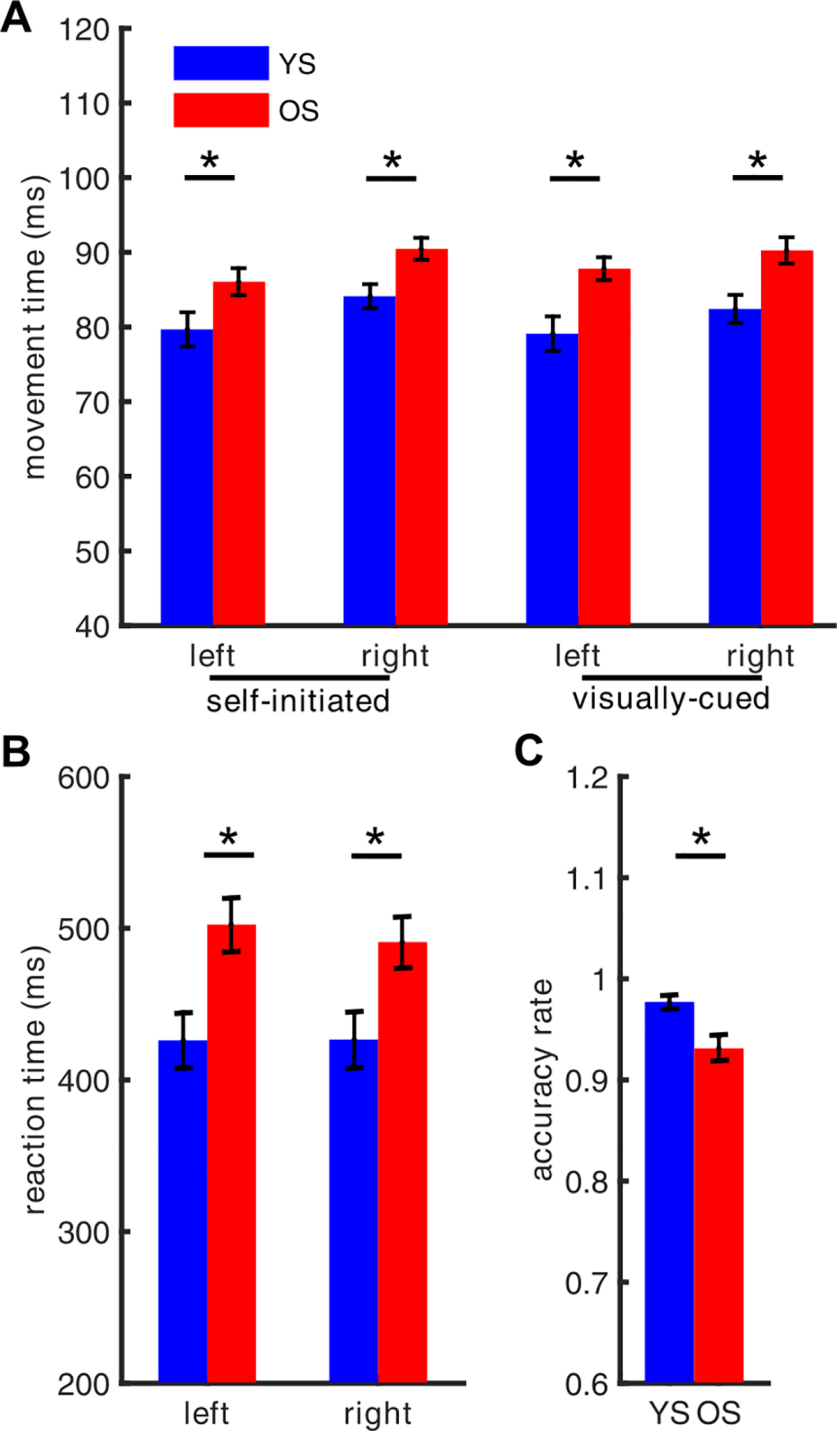
(A) Movement time in the visually-cued and the self-initiated tapping condition for younger (YS, blue) and older (OS, red) participants. (B) Reaction time and (C) accuracy rate (averaged over all index finger tappings) in the visually-cued tapping condition of younger (YS, blue) and older (OS, red) subjects. Stars above the bars denote a statistically significant (p < 0.05) between-group difference.

Furthermore, we found that the movement time was significantly longer in older subjects than in younger subjects in both conditions (see Fig 2A, t (166) = 5.5839, p < 0.0001). In addition, in the visually-cued condition, the reaction time was significantly longer in older subjects than in younger subjects (see Fig 2B, t (82) = 3.9442, p < 0.0001).

The mean response accuracy in the visually-cued condition for older participants (93.13% ± 5.69%) and for younger subjects (97.7% ± 2.22%) was greater than 90%, consistent with the instructions. However, as possibly expected, accuracy was lower for the older participants (see Fig 2C, t (40) = -3.2329, p = 0.0025). In summary, we found an age-dependent slow-down and a diminished accuracy of the execution of the motor task.

### Electrophysiological results in the visually-cued tapping condition

#### Phase locking in the δ-θ frequency band

**Fig 3:**
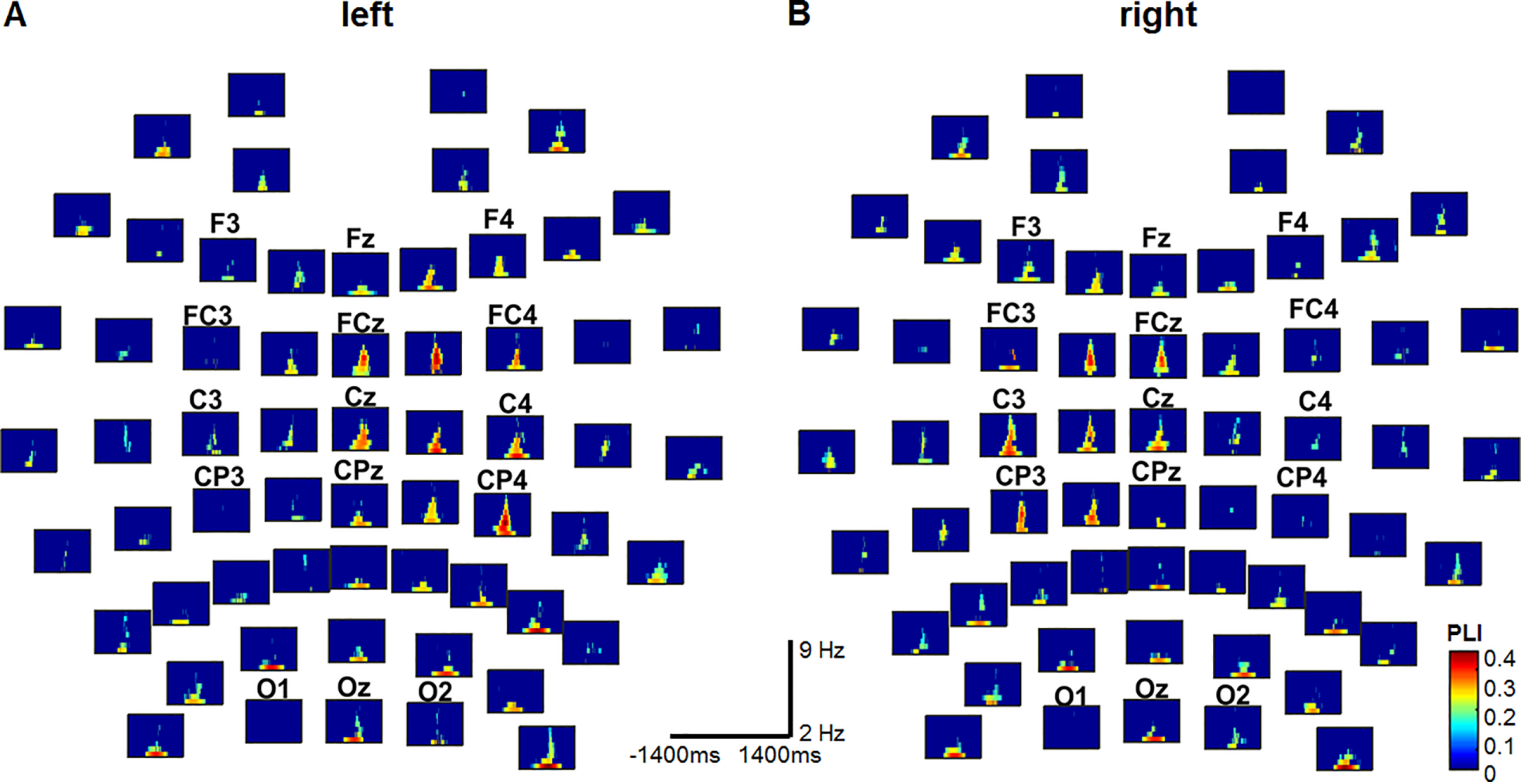
Phase-locking index in older subjects at all 61 electrodes in the visually-cued tapping condition with the left (A) or right index finger (B). At electrodes where the PLIs were not significantly larger than those of the corresponding baselines, the panels were left blank (dark blue) as in [9]. In all panels, the horizontal axis is the time axis in the interval [-1400, 1400] ms, and the vertical axis is the frequency axis in the band 2–9 Hz. The onset of the movement, as determined by the accelerometer, is at time t = 0. The color bar at the bottom right indicates the value of the PLI in the individual panels.

In Fig 3, the PLIs obtained from the older participants are displayed for all 61 channels for left (left) and right (right) index finger tapping. The small panels are organized according to the location of the electrodes on the scalp. In all of them, the horizontal axis is the time axis in the interval [-1400, 1400] ms, and the vertical axis is the frequency in the band 2–9 Hz. No significant PLI occurred at frequency values greater than 9 Hz. We assigned t = 0 to the onset of the movement. The color bar at the bottom right indicates the PLI value in the individual panels. As in [9], the panels with no significant PLI were automatically left blank (dark blue). PLIs significantly greater than that of the baseline can clearly be discerned at the central electrodes in the low frequency band (2–7 Hz) near movement onset in both conditions and for both hands. These electrodes, i.e., FC1-FC2, FC3-FC4, FCz, C1-C2, C3-C4, Cz, CP1-CP2, CP3-CP4, and CPz, are located closest to the motor regions (Fig 3). The EEG at them has been shown to exhibit significant PLI in younger subjects too (cf. [9]). In addition to these electrodes, we found significant phase locking at the frontal electrodes, i.e., F1-F2, F3-F4, and Fz, both in older and younger subjects. Additional significant PLIs occurred at the electrodes lying above the occipital regions (Fig 3).

**Fig 4:**
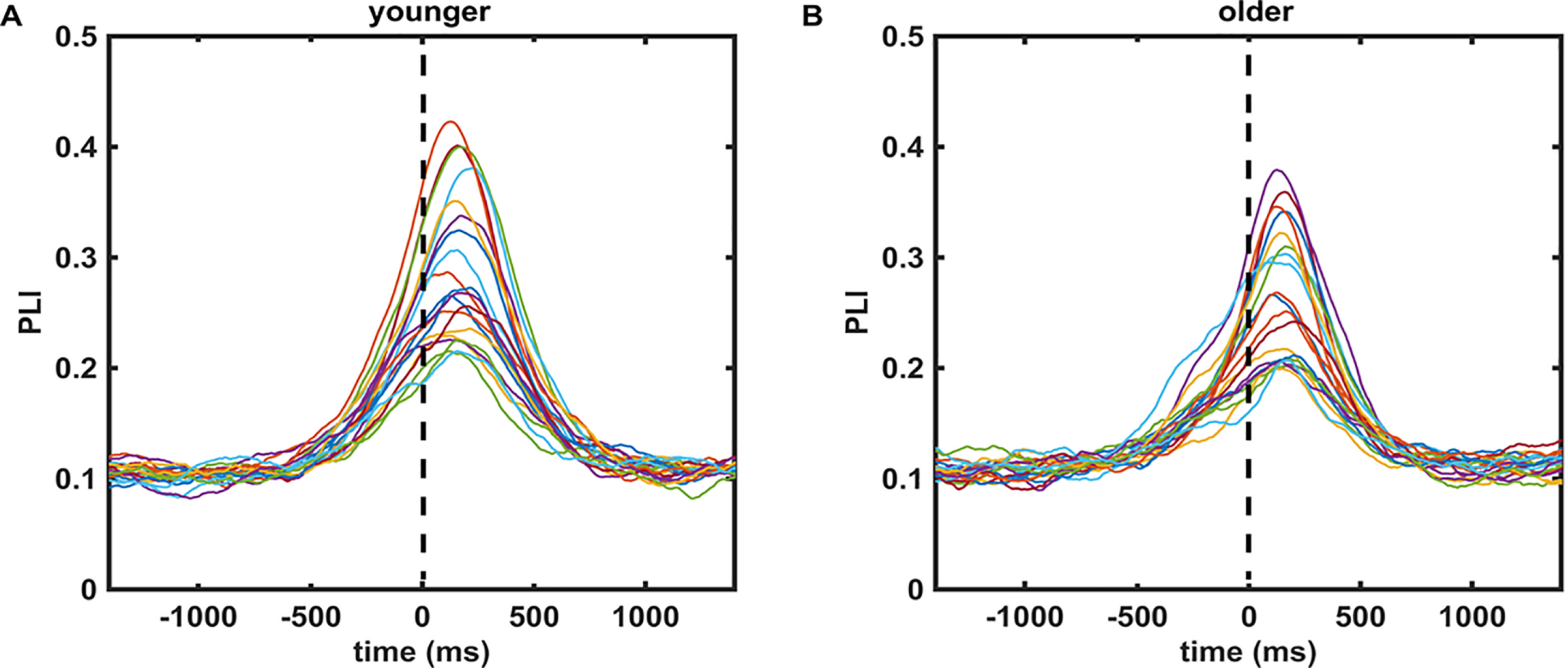
Shows the averaged PLI over the δ-θ frequency band for 20 electrodes of interest in both younger (A) and older (B) subjects. Each colored line represents the averaged PLI for one electrode. The same color coding of the 20 electrodes was used for the left and right panel.

We tested whether the strength and length of phase locking was significantly different in older compared to younger subjects. For this, we compared the values of the mean PLI over the δ-θ frequency band for each of the 20 electrodes listed above at each time point in the time interval [-1400 1400] ms (cf. averaged PLI curves in Fig 4). No time interval in which a significant difference occurred was found. Then we tested the maximum value of the mean PLIs in the δ-θ frequency band. It did not show any significant difference between older and younger subjects for either hand.

In summary, we found that the strong phase locking in the δ-θ frequency band around movement onset, being strongest at the electrodes above the motor cortex contralateral to the moving hand, reported for younger subjects [9], was unaffected by age even though the accuracy and timing with which the older subjects performed the task was worse. The results thus suggest that the increased variability in motor execution in older subjects was not driven or caused by differences in phase locking in the δ-θ frequency band.

#### Post-movement β amplitude

To further investigate the neural correlates of the reduced accuracy of movement and reaction time of older subjects, we next investigated the second characteristic of neural oscillations in the time-frequency domain: their amplitude.

**Fig 5:**
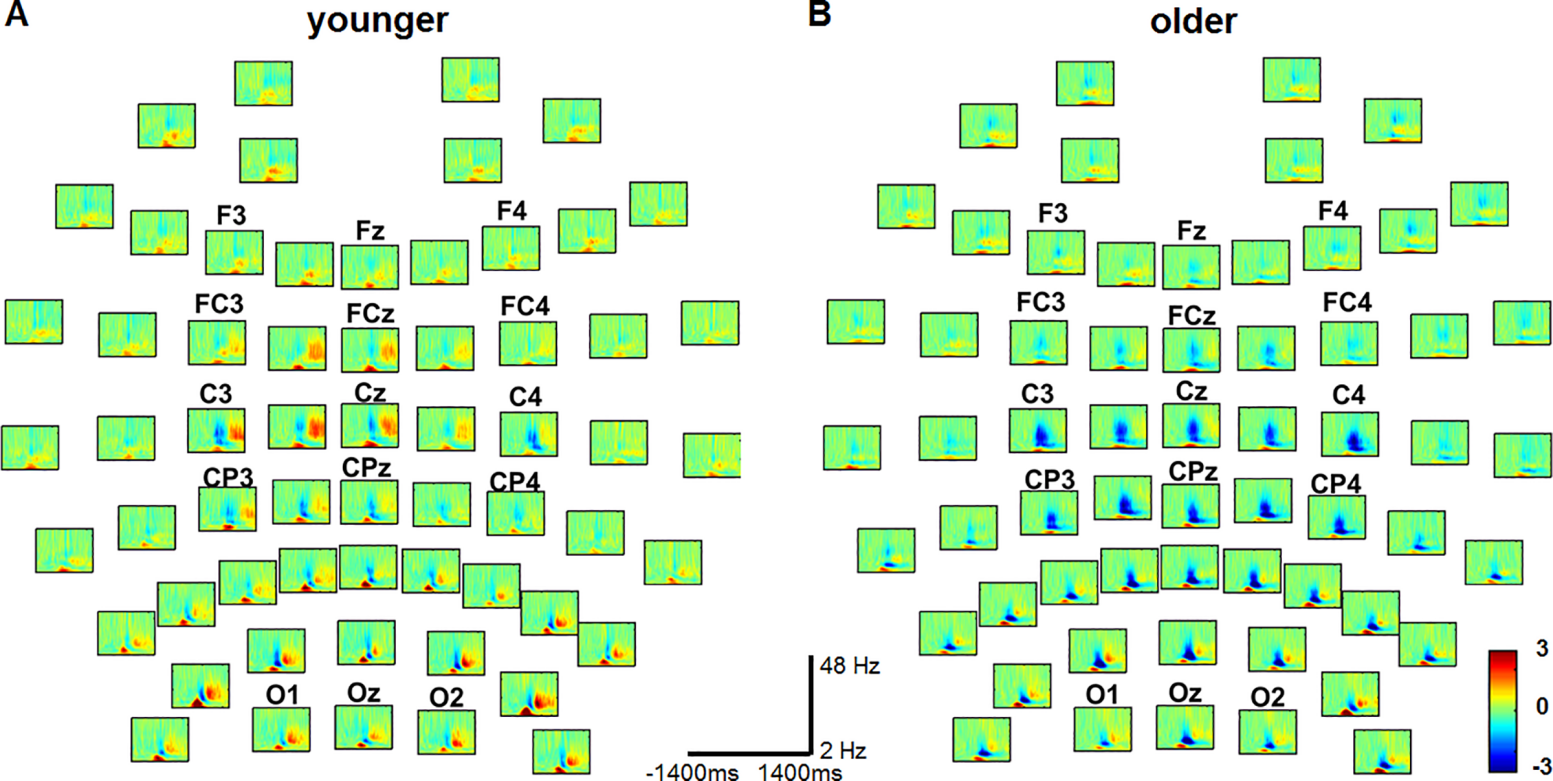
Amplitude (power spectrum) at all 61 electrodes in the visually-cued right-hand tapping condition in younger (A) and older subjects (B). In all panels, the horizontal axis is the time in the interval [-1400, 1400] ms, and the vertical axis is frequency in the range 2–48 Hz. The onset of the movement, as determined by the accelerometer, is at t = 0. The color bar at the bottom right indicates the value of the amplitude (power) at a given frequency in the individual panels.

Fig 5 shows the amplitude dynamics in the frequency domain 2 Hz to 48 Hz in both younger and older subjects for all 61 electrodes, exemplified for right index finger button presses. The small panels are arranged according to the location of the electrodes on the scalp. In the panels, the horizontal axis is the time (from -1400 ms to 1400 ms) and the vertical axis is the frequency. We found a significant decrease in amplitude mainly in the α and β frequency band (ERD) starting already before movement onset and a significant increase in amplitude after termination of the movement (ERS) in the β frequency band in younger subjects. In older subjects, we also observed a significant decrease in amplitude in the α and β frequency band, which started before movement onset, but the increase in amplitude after movement termination was not as high as the corresponding ERS measured in younger subjects (see Fig 5, ERD in blue, ERS in red).

S1 Fig (Supplementary Materials) shows an example of significant ERD and ERS in β amplitude relative to the baseline at electrodes lying above the primary motor cortex contralateral to the moving hand (C3) and the supplementary motor area (FCz) in the visually-cued tapping condition, when button presses were performed with the right index finger, for both younger and older subjects.

**Fig 6:**
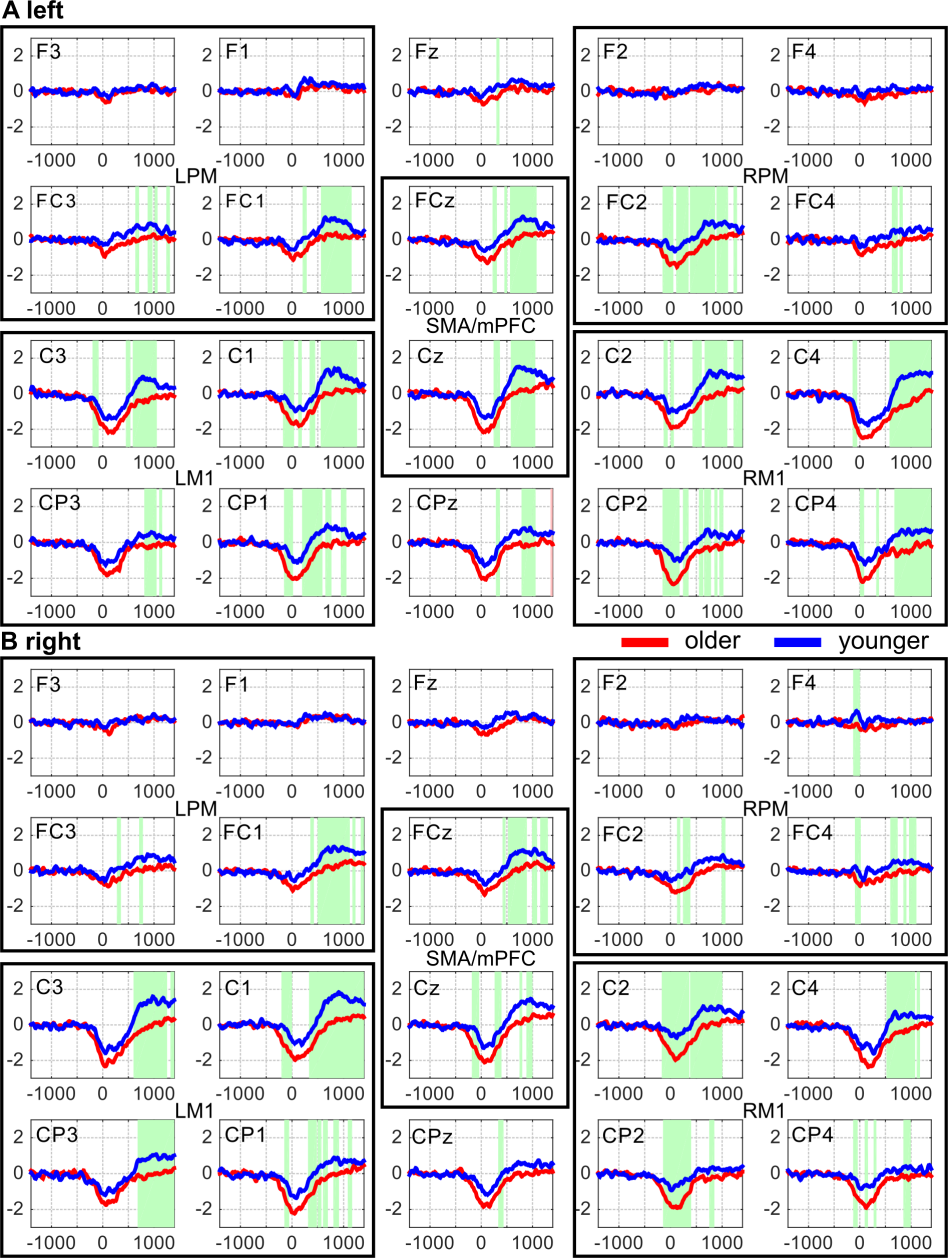
Amplitude averaged over the β (13–30 Hz) frequency band in visually-cued left (A) and right index finger tapping (B) in both younger (blue lines) and older (red lines) subjects at the electrodes of interest that label the panels. The horizontal axis is the time in the interval [–1400, 1400] ms. The vertical axis shows the size of the amplitudes. In the time intervals marked with green, the amplitudes in the younger subjects are significantly higher (p<0.0085, FDR corrected) than those in the older subjects. LPM and RPM denote left and right premotor cortex, LM1 and RM1 left and right primary motor cortex, respectively. SMA stands for the supplementary motor area. Please note that FCz also detects activity from neural populations in the medial prefrontal cortex (mPFC).

To test whether the amplitudes measured in the two age groups significantly differed, we averaged the amplitude within each of the four frequency bands (δ-θ, α, β, and low γ). The average amplitudes were tested for time intervals of significant differences between older and younger subjects at the 20 electrodes of interest, separately. We mainly observed significant differences in the β frequency band (Fig 6).

In the α frequency band, these time intervals were only present at a couple of electrodes and only for a very short time interval around movement onset. No significant changes were found in the δ-θ and low γ frequency bands. We therefore carried out further analyses on the amplitudes in the β frequency band, only. In this frequency band, we observed a significant decrease of the amplitude after termination of the movement in older subjects compared to that in younger subjects (see Fig 6, p < 0.0085, FDR corrected). The blue and red curves indicate the averaged β amplitude in younger and older subjects, respectively. In the green time intervals, mean β amplitudes in the two age groups differed significantly. The time intervals of significant differences started around 500 ms after movement onset and lasted until around 1000 ms after movement onset or even longer. The longest time intervals of this kind were observed at the electrodes that capture neural activity from the *supplementary motor area (SMA) and the medial prefrontal cortex (mPFC)* and *bilaterally from the primary motor cortex (M1)*.

At some electrodes, a small time interval of significant difference in the mean β amplitude also occurred around movement onset (see Fig 6).

In summary, we found significantly lower post-movement β amplitudes in the older subjects than in the younger ones during visually-cued movements. This finding was most pronounced in SMA/mPFC and M1 bilaterally.

To verify that the difference in β amplitude between younger and older subjects is statistically robust, we used a three-way (groups x electrodes x hands) ANOVA on the averaged β amplitude over the time interval 500 ms to 1250 ms. We found a main effect of both group (F (1, 40) = 401.26, p < 0.0001) and electrodes (F (19, 1560) = 7.74, p < 0.0001). There was also a significant interaction effect (F (19, 1560) = 6.24, p < 0.0001) of groups and electrodes.

#### Correlation between averaged post-movement β amplitude and behavioral performance

Finally, we tested whether differences in the mean β amplitude between younger and older subjects were linked to behavioral performance. As the main differences in the mean β amplitude between the two age groups occurred in the time interval 500 ms to 1250 ms after movement onset, we averaged the mean β amplitude over this time interval and obtained a single β amplitude value at each electrode for each subject. Next, we tested whether this post-movement β amplitude at the 20 electrodes of interest correlated with the subjects' behavioral performance. We used the reaction time, the movement time and the accuracy rate as behavioral measures.

**Fig 7:**
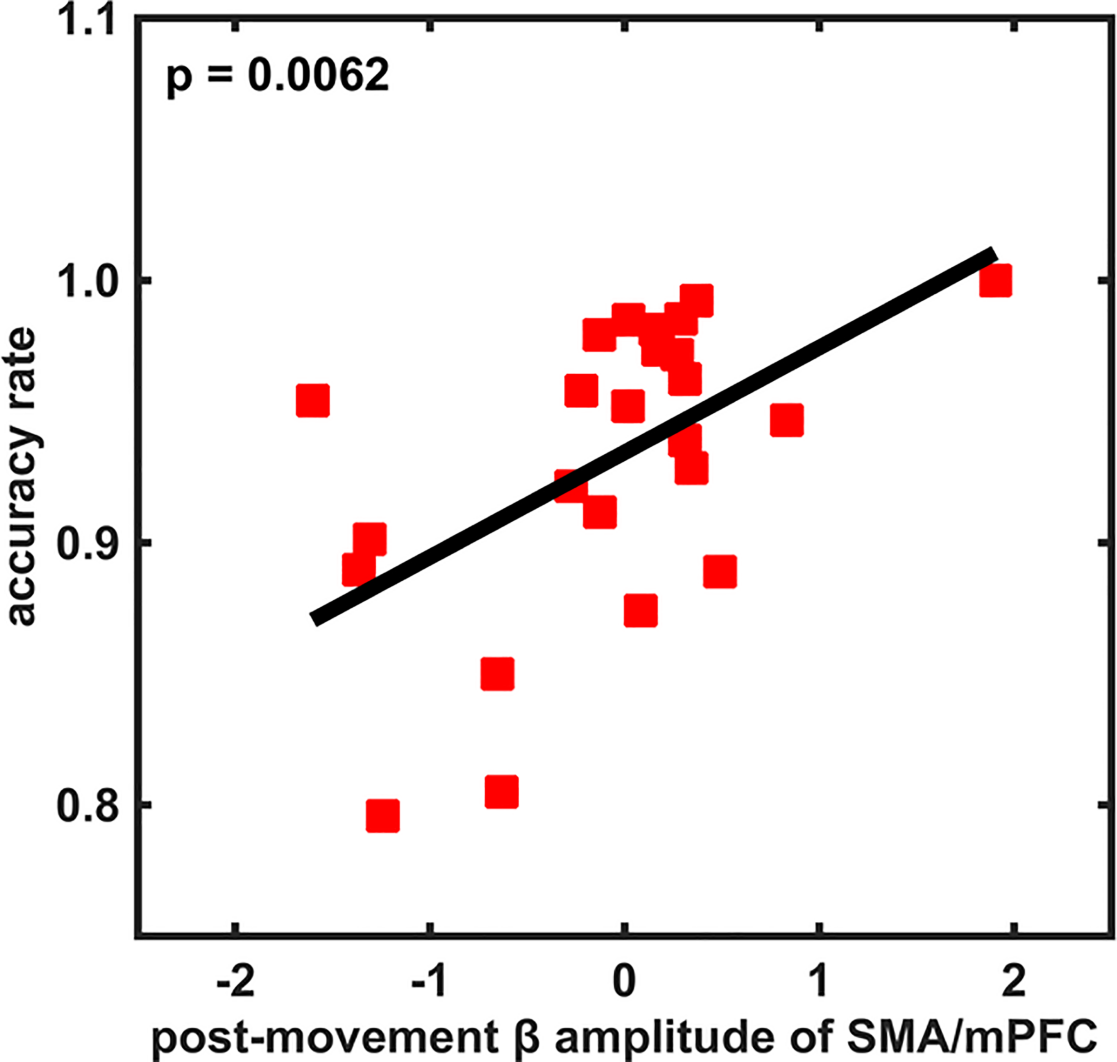
Correlation between the time-averaged post-movement β amplitude and the accuracy rate (averaged over all index finger tappings) in the visually-cued tapping condition in older subjects. The horizontal axis in each panel is the β amplitude averaged in the time interval [500, 1250] ms, and the vertical axis is the accuracy rate.

We did not find any correlation between post-movement β amplitude and reaction or movement time at any of the aforementioned electrodes in either of the age groups. However, in the group of older subjects, we observed a significant positive correlation between accuracy rate and post-movement β amplitude at FCz (i.e., above SMA and mPFC) (Pearson, r (24) = 0.5422, p = 0.0062, Fig 7). Furthermore, we observed that those older subjects who attained an accuracy rate as good as that of the younger subjects had an equally high post-movement β amplitude as the younger subjects.

Since we also observed small time intervals of significantly smaller mean β amplitude around movement onset in older subjects, we also tested if there was a correlation between the mean β amplitude, averaged in the time interval from -100 ms to 200 ms, and the behavioral performance of the individual subjects. We did not find any correlation between this averaged β amplitude around movement onset and the reaction or movement time, or the accuracy rate.

In summary, our results indicate that the higher the post-movement β amplitude was in the participants performing visually-cued movements, the more accurately they carried out the motor task.

### Electrophysiological results in the self-initiated tapping condition

In S2 Fig (Supplementary Materials), the PLIs obtained from the older participants are displayed for all 61 channels for left (A) and right (B) self-initiated index finger tapping. Significant PLIs compared to baseline are most pronounced at the electrodes above the brain regions contralateral to the moving index finger. In accordance with the results obtained from the visually-cued tapping condition, there is no significant difference in PLI at the 20 electrodes of interest between younger and older subjects in the self-initiated tapping condition.

To test whether the amplitude shows differences between younger and older subjects, we averaged the amplitude in the δ-θ, α, β and γ frequency bands and tested the averages separately. We found significantly smaller β amplitude in the older participants than in the younger ones. This finding mainly applied to the electrodes situated above SMA/mPFC and M1 bilaterally in the time interval [500,1250] ms after movement onset (see S3 Fig). This is consistent with what was seen in the visually-cued condition. However, no correlation was found between the post-movement β amplitude in SMA/mPFC and the movement time in both younger and older subjects.

## Discussion

We investigated age-related changes in phase locking and amplitude (power density) of EEG in the frequency domain at the motor regions of the brain in younger and older subjects in order to unravel neural mechanisms underlying age-related increased variability in motor output. At the behavioral level, we found significantly longer reaction and movement times and a lower accuracy rate of the tasks performed by older adults. Similar age-related behavioral effects have also been reported by other authors [2, 24, 25].

### Phase locking in younger and older subjects

Reflecting neural processes, phase locking of EEG was most pronounced over the motor cortex in the δ-θ frequency band around movement onset. This was independent of whether the movement was performed internally or externally driven. No significant age-related differences were discerned, neither for the left nor for the right (dominant) hand.

As self-initiated and visually-cued movements are of two different types, the δ-θ PLIs differ—as expected—in the two conditions. In both conditions, a strong PLI was found at the electrodes located above the motor regions while an additional strong PLI in the occipital regions occurred in the visually-cued condition only. This PLI might have arosen due to external cues.

Moreover, we did not find evidence that phase locking was related to speed or accuracy of the tasks being performed. Since the older subjects were able to initiate the motor action, i.e. trigger movement execution, the same way as the younger ones, phase locking seems to be a general marker of movement initiation, rather than an indicator of the actual movement performance. This is in accordance with previous results in younger subjects [9].

Previous EEG studies on effects of aging in the human motor network mainly focused on changes in event-related potentials or the power spectrum (amplitude) of the signals. Hence, data on age-related local phase locking differences are scarce. Results obtained from studies analyzing age-related changes in tasks with a higher cognitive load than that in our present study (e.g. [26, 27]) are consistent with ours to the extent that phase synchrony in lower frequency bands is assumed to constitute an important mechanism for organizing neural ensembles to perform a task [28].

In general, local phase locking reflects the same timing of activity of many neurons, or a periodic timing of the same neurons, hence true synchronization [29]. Lee and colleagues postulated that θ oscillations provide a mechanism for controlling the interaction between neurons in different brain regions [30]. Furthermore, [28] showed, albeit in the rat, that θ oscillations play a part in the neural coordination during motor activity, in particular motor preparation and action. These authors demonstrated a close (functional) connection between θ oscillation and layer-dependent firing of cortical neurons (at high and low β frequencies). Their findings, at least indirectly, support our results concerning the role of δ-θ oscillations as indicators of human motor actions.

Advancing adult age could lead to increased neural noise [31, 32] which might result in increased movement variability. However, we showed in the current study that the changes in movement variability that we observed in older subjects were not reflected by local phase locking in the motor system. These results indicate that temporal precision of neural synchronization in the motor system appears to maintain in older subjects [27]. The high level local phase locking in older subjects might reflect a loss of complexity of the neural response [33]. In other words, neural signal processing in older subjects requires more efforts, especially when loss of neural substance happens. In addition, it should be noted that the increased variability of movement in older subjects most likely results from complex mechanisms including both muscular and neural changes. Thus variability of movement could also be enhanced due to changes in the muscular system [5].

### Post-movement β amplitude and motor accuracy

We also found significantly lower post-movement β amplitudes in the older subjects than in the younger ones during both self-initiated and visually-cued movements, especially in SMA/mPFC and bilateral M1. These results are consistent with those of earlier reports [12, 34, 35].

The study [12] on event-related potentials, for instance, suggested that a functional dysregulation of the contralateral motor cortex contributes to longer reaction times in older individuals, and mainly happens during response generation. The authors of [35] found that, during passive ankle movements, the lower amplitude and long latency of the N1 component of the evoked EEG potential correlated with a delayed response in older subjects. Labyt and colleagues [34]made use of a visually-guided motor task to assess the effect of aging on the spatio-temporal patterns of cortical activity. They also found a higher and wider event-related synchronization in younger adults than in older ones when both younger and older participants performed the same motor action.

Furthermore, [36] suggested that post-movement β rebound (PMBR) shows an effect of age in that PMBR was significantly diminished in young children. The authors of this study based their claim on a comparative MEG study in which they measured visually-cued motor responses in both children and adults. They also hypothesized that PMBR may reflect an age-dependent inhibitory process in the primary motor cortex, which is part of the normal development process.

In agreement with that, previous studies suggested that PMBR, or event-related synchronization (ERS), is related to a state of cortical "deactivation" of locally restricted motor networks [37], to “re-setting” of the motor networks involved in the task [38], or to movement-related somatosensory processing [39].

Importantly, we observed that the post-movement β amplitude (power) in FCz positively correlated with the accuracy rate in the older subjects, meaning that the higher the post-movement β amplitude (power) was in the participants carrying out visually-guided movements, the more accurate the motor act was performed. In other words, older subjects who had an accuracy rate in the same range as the younger ones exhibited the same strength in post-movement β power.

We did not find any correlation between the post-movement β amplitude and the accuracy rate in the younger subjects. As the task we applied was a very simple visually-guided task, the younger subjects already had a very high accuracy rate. This might hint at a potential ceiling effect of the performance in the younger subjects.

Oscillations at FCz electrodes detect activity from neural generators not only in the SMA, but also in the medial prefrontal cortex (mPFC), which includes the preSMA and the dorsal anterior cingulate cortex (ACC) [40]. The area mPFC, especially ACC, has been extensively implicated in the monitoring of cognitive-motor interactions [40–42]. This relationship of the mPFC to task performance suggests that the post-movement β power at FCz may be indicative of impaired cognitive control and therefore explain the observed decrease in accuracy of the visually-guided actions of older subjects. Several lines of evidence support this hypothesis. The mPFC consistently shows task-induced deactivation (TID) of its cortical neurons irrespective of the task being performed [43]. Numerous studies have shown an age-related modulation of the spatial extent and the magnitude of TID. Lustig and colleagues [16], for instance, found decreased deactivation in older adults relative to younger subjects in the mPFC during a semantic classification task (compared to rest). Older adults showed a flattened and slowed-down time course of blood oxygen level-dependent (BOLD) fMRI response when compared to younger subjects. These results were further confirmed by Grady et al. [43] who investigated a population spanning from 20–87 years and found an age-related linear increase in BOLD activity during a fixation baseline (relative to a task) in medial default mode network (DMN) regions: Older adults showed an increase in activity of brain regions not traditionally recruited during the performance of the task, such as the mPFC, whereas they showed decreased activation in task-relevant areas when compared to younger subjects. Thus, data suggest that an age-associated reduced cognitive control may result from a reduction in task-induced deactivation of the respective cortical neurons [44].

In summary, our study shows that phase and amplitude play different roles in the control of how accurately a motor act is performed. In particular, phase locking in the δ-θ frequency band on the one hand seems to be a general movement-related phenomenon, which does not seem to have an influence on the actual performance of the movement. The decreased post-movement β amplitude in the mPFC of older subjects might, on the other hand, be related to an impaired deactivation of its local neural populations. This reflects an age-related effect on the cognitive control of stimulus-induced motor tasks associated with impaired motor accuracy.

## Supporting information

S1 Fig Changes in amplitude (power spectrum) relative to baseline at electrodes situated above the primary motor cortex contralateral to the moving hand (C3, panels in upper row) and the supplementary motor area and the medial prefrontal cortex (FCz, panels in lower row) in the visually-cued tapping condition performed with the right index finger in the younger (A) and the older (B) subjects. In A, B and C, the horizontal axis is the time in the interval [–1400, 1400] ms. In A and B, the vertical axis is the frequency in the band 2–48 Hz, in C, the averaged amplitude in the β (13–30 Hz) frequency band. The solid lines on the time axis in C indicate the time intervals in which the ERD was significant in the older (red, OS) and the younger (blue, YS) subjects, the dashed lines those in which ERS was significant in the older (red, OS) and the younger (blue, YS) subjects.
(TIF)

S2 Fig Phase-locking index (PLI) in older subjects at all 61 electrodes in the self-initiated tapping condition with the left (A) or right index finger (B). At electrodes where the PLIs were not significantly larger than those of the corresponding baselines, the panels were left blank (dark blue) as in [9]. In all panels, the horizontal axis is the time axis in the interval [–1400, 1400] ms, and the vertical axis is the frequency axis in the band 2–9 Hz. The onset of the movement, as determined by the accelerometer, is at time t = 0. The color bar at the bottom right indicates the value of the PLI in the individual panels.
(TIF)

S3 Fig Amplitude averaged over the β (13–30 Hz) frequency band in self-initiated left (A) and right index finger tapping (B) in both the younger (blue lines) and the older (red lines) subjects at the electrodes of interest that label the panels. The horizontal axis is the time in the interval [–1400, 1400] ms. The vertical axis shows the size of the amplitudes. In the time intervals marked with green, the amplitudes in the younger subjects are significantly higher (p<0.05, FDR corrected) than those in the older subjects. LPM and RPM denote left and right premotor cortex, LM1 and RM1 left and right primary motor cortex, respectively. SMA stands for the supplementary motor area. Please note that FCz also detects activity from neuronal populations in the medial prefrontal cortex (mPFC).
(TIF)
