# Supplementary figures and images for: Age-related changes in oscillatory power affect motor action
Source: PLoS One. 2017 Nov 27;12(11):e0187911. doi: 10.1371/journal.pone.0187911 (PMC5703531; doi:10.1371/journal.pone.0187911)

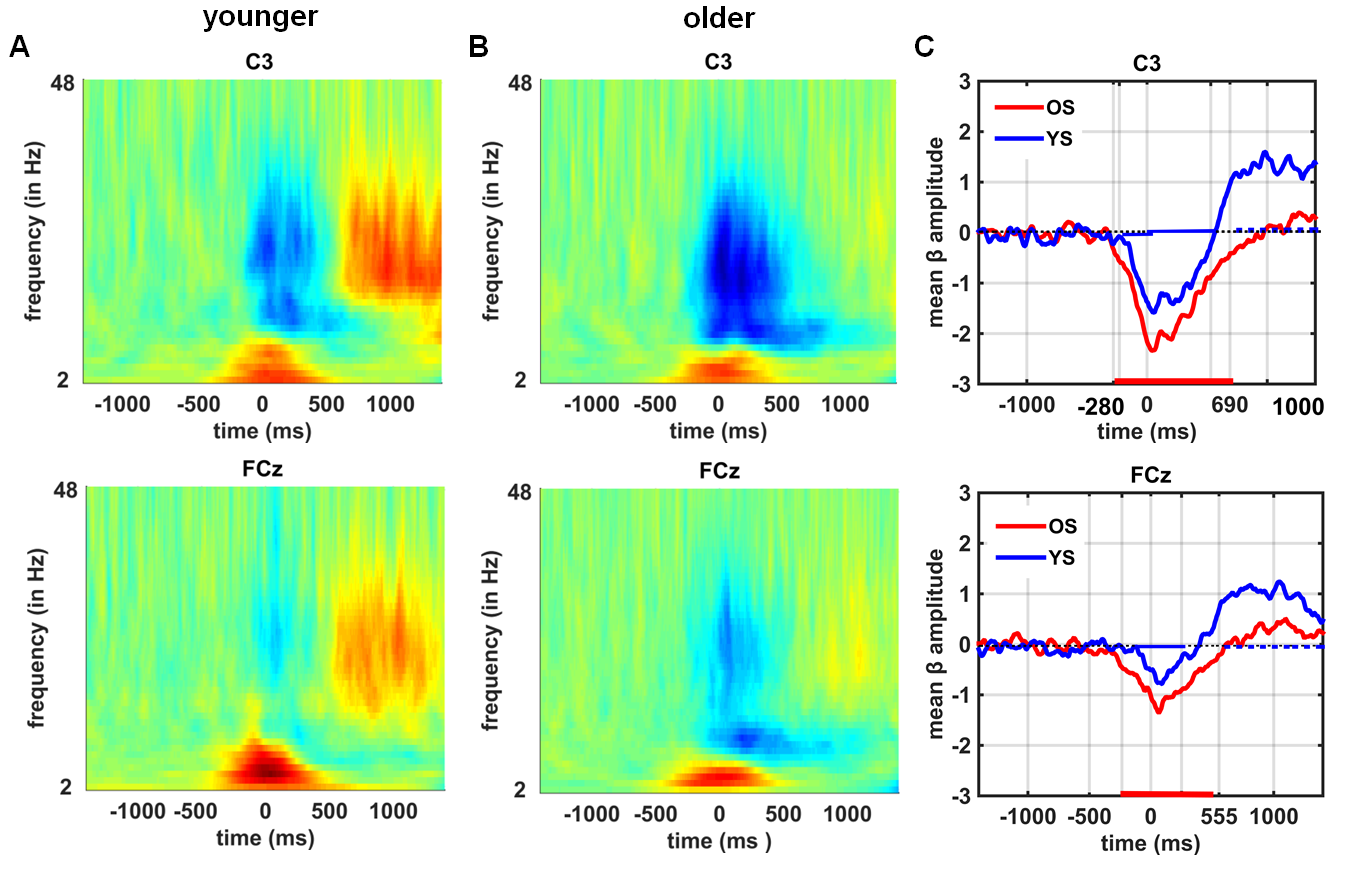

Supplement: S1 Fig — Changes in amplitude (power spectrum) relative to baseline at electrodes situated above the primary motor cortex contralateral to the moving hand (C3, panels in upper row) and the supplementary motor area and the medial prefrontal cortex (FCz, panels in lower row) in the visually-cued tapping condition performed with the right index finger in the younger (A) and the older (B) subjects. In A, B and C, the horizontal axis is the time in the interval [–1400, 1400] ms. In A and B, the vertical axis is the frequency in the band 2–48 Hz, in C, the averaged amplitude in the β (13–30 Hz) frequency band. The solid lines on the time axis in C indicate the time intervals in which the ERD was significant in the older (red, OS) and the younger (blue, YS) subjects, the dashed lines those in which ERS was significant in the older (red, OS) and the younger (blue, YS) subjects. (TIF) [file pone.0187911.s001.tif]

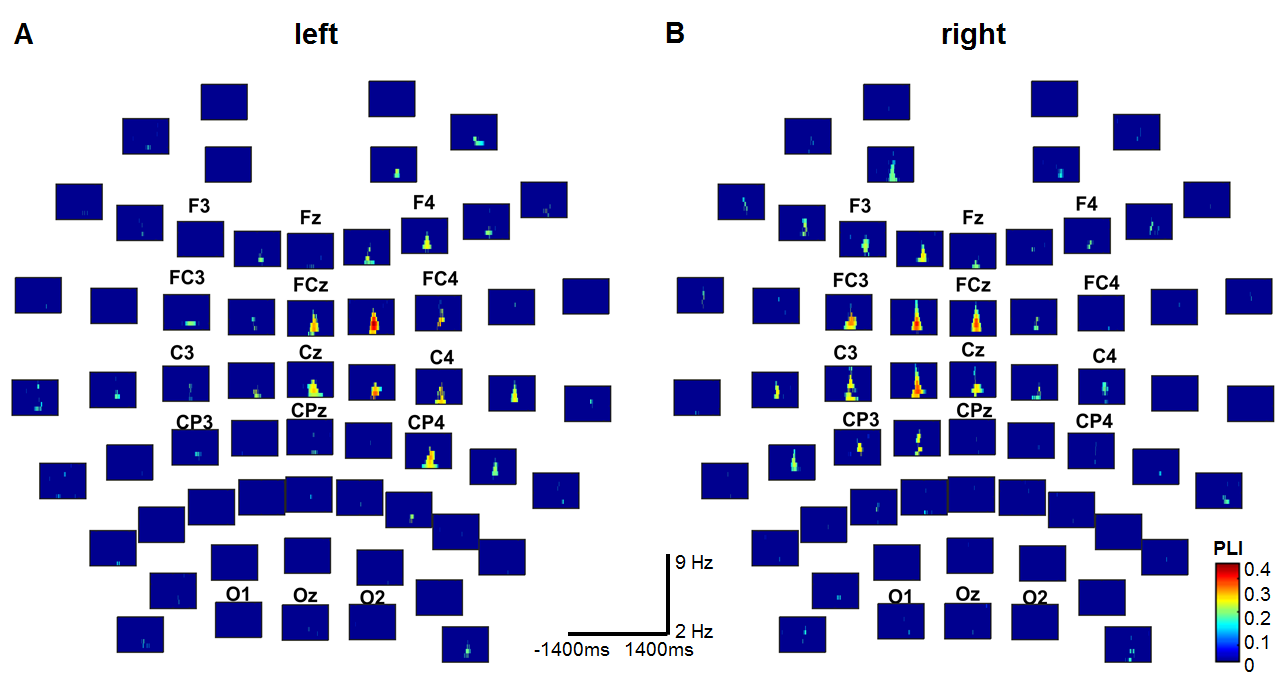

Supplement: S2 Fig — Phase-locking index (PLI) in older subjects at all 61 electrodes in the self-initiated tapping condition with the left (A) or right index finger (B). At electrodes where the PLIs were not significantly larger than those of the corresponding baselines, the panels were left blank (dark blue) as in [9]. In all panels, the horizontal axis is the time axis in the interval [–1400, 1400] ms, and the vertical axis is the frequency axis in the band 2–9 Hz. The onset of the movement, as determined by the accelerometer, is at time t = 0. The color bar at the bottom right indicates the value of the PLI in the individual panels. (TIF) [file pone.0187911.s002.tif]

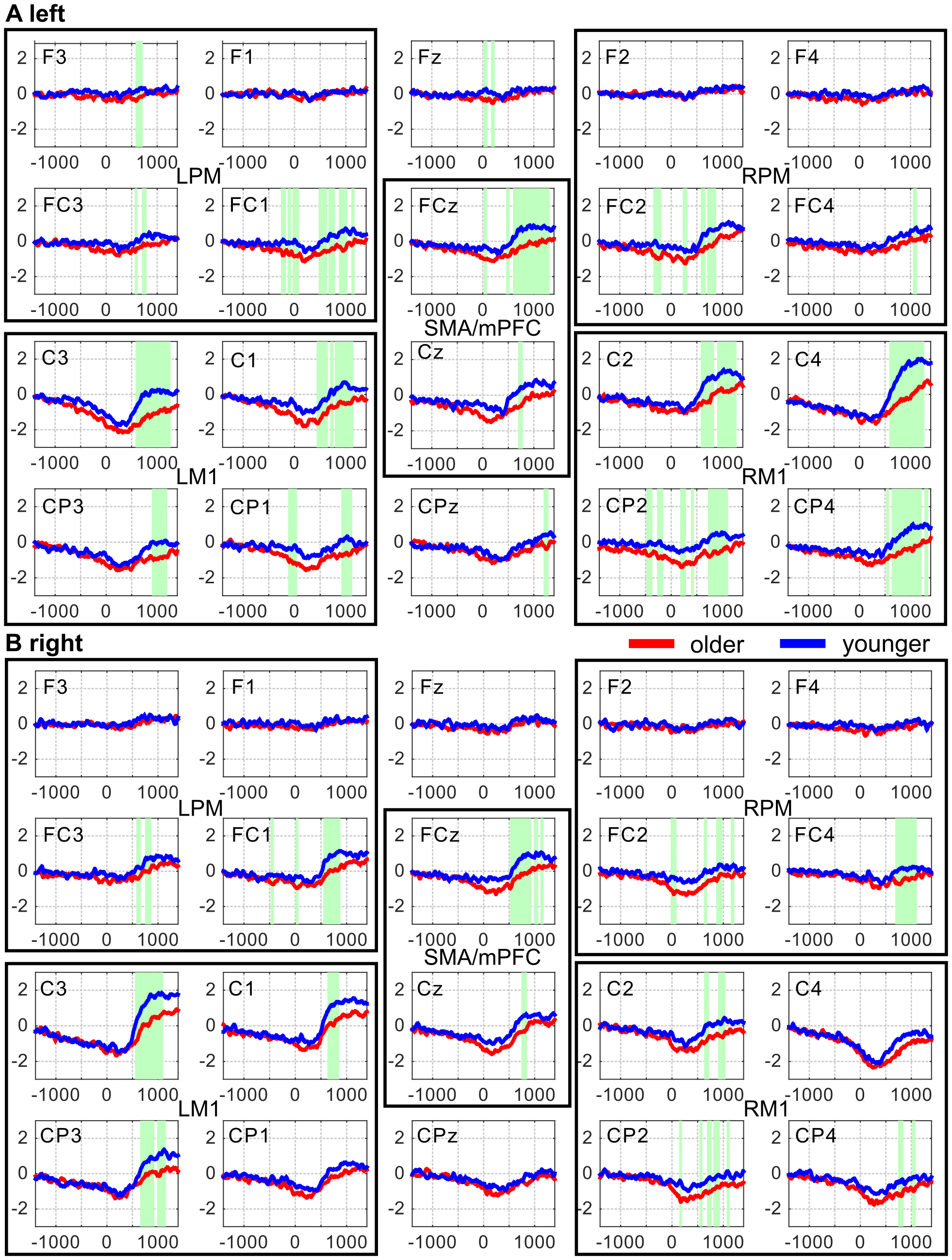

Supplement: S3 Fig — Amplitude averaged over the β (13–30 Hz) frequency band in self-initiated left (A) and right index finger tapping (B) in both the younger (blue lines) and the older (red lines) subjects at the electrodes of interest that label the panels. The horizontal axis is the time in the interval [–1400, 1400] ms. The vertical axis shows the size of the amplitudes. In the time intervals marked with green, the amplitudes in the younger subjects are significantly higher (p<0.05, FDR corrected) than those in the older subjects. LPM and RPM denote left and right premotor cortex, LM1 and RM1 left and right primary motor cortex, respectively. SMA stands for the supplementary motor area. Please note that FCz also detects activity from neuronal populations in the medial prefrontal cortex (mPFC). (TIF) [file pone.0187911.s003.tif]
